# Supraphysiologic doses of 17β-estradiol aggravate depression-like behaviors in ovariectomized mice possibly via regulating microglial responses and brain glycerophospholipid metabolism

**DOI:** 10.1186/s12974-023-02889-5

**Published:** 2023-09-07

**Authors:** Ming Li, Jing Zhang, Wendi Chen, Shuang Liu, Xin Liu, Yunna Ning, Yongzhi Cao, Yueran Zhao

**Affiliations:** 1 Center for Reproductive Medicine, Shandong University, Jinan, 250012 Shandong China; 2 Key Laboratory of Reproductive Endocrinology of Ministry of Education, Shandong University, Jinan, 250012 Shandong China; 3 National Research Center for Assisted Reproductive Technology and Reproductive Genetics, Shandong University, Jinan, 250012 Shandong China

**Keywords:** 17β-Estradiol, Estrogen replacement therapy, Menopausal syndrome, Depression, Microglial, Glycerophospholipid metabolism

## Abstract

**Background:**

17β-Estradiol (E2) is generally considered neuroprotective in humans. However, the current clinical use of estrogen replacement therapy (ERT) is based on the physiological dose of E2 to treat menopausal syndrome and has limited therapeutic efficacy. The efficacy and potential toxicity of superphysiological doses of ERT for menopausal neurodegeneration are unknown.

**Methods:**

In this study, we investigated the effect of E2 with a supraphysiologic dose (0.5 mg/kg, sE2) on the treatment of menopausal mouse models established by ovariectomy. We performed the open field, Y-maze spontaneous alternation, forced swim tests, and sucrose preference test to investigate behavioral alterations. Subsequently, the status of microglia and neurons was detected by immunohistochemistry, HE staining, and Nissl staining, respectively. Real-time PCR was used to detect neuroinflammatory cytokines in the hippocampus and cerebral cortex. Using mass spectrometry proteomics platform and LC–MS/ MS-based metabolomics platform, proteins and metabolites in brain tissues were extracted and analyzed. BV2 and HT22 cell lines and primary neurons and microglia were used to explore the underlying molecular mechanisms in vitro.

**Results:**

sE2 aggravated depression-like behavior in ovariectomized mice, caused microglia response, and increased proinflammatory cytokines in the cerebral cortex and hippocampus, as well as neuronal damage and glycerophospholipid metabolism imbalance. Subsequently, we demonstrated that sE2 induced the pro-inflammatory phenotype of microglia through ERα/NF-κB signaling pathway and downregulated the expression of cannabinoid receptor 1 in neuronal cells, which were important in the pathogenesis of depression.

**Conclusion:**

These data suggest that sE2 may be nonhelpful or even detrimental to menopause-related depression, at least partly, by regulating microglial responses and glycerophospholipid metabolism.

**Supplementary Information:**

The online version contains supplementary material available at 10.1186/s12974-023-02889-5.

## Introduction

The climacteric period is commonly known as menopause, marking the end of the reproductive period for most women [1]. With the increased social pressure and the accelerated pace of life, the incidence of menopausal depression in women tends to be high [2]. Generally, depression is a broad and heterogenous diagnosis, with depressed mood and/or loss of pleasure in activities as the main diagnostic characteristics [3].

Many studies have revealed that neuroinflammation, triggered by the activation of microglia, is closely related to the pathogenesis of depression [4]. Microglia are important immune cells in the central nervous system (CNS), playing an important role in the occurrence and development of depression [5]. Under normal physiological conditions, microglia are in a quiescent state, monitoring CNS homeostasis. In response to stresses or abnormal neuronal activities, the morphology and functions of microglia are rapidly changed, presenting an activated state. According to the “gliocentric theory,” stress-induced inflammation resulting from microglia activation may trigger a cascade of glial dysfunctions that supports the development of depressive disorders [6].

Evidence suggests that the endogenous cannabinoid system is involved in the pathophysiology of depression [7]. Endocannabinoids are signaling lipids that activate cannabinoid receptors in the CNS and peripheral tissues. Cannabinoid receptors are divided into two classes, i.e., cannabinoid receptor 1 (CB1) and cannabinoid receptor 2 (CB2). The CB1 receptors in the brain are mainly distributed in presynaptic axons and nerve endings [8]. Certain genetic polymorphisms in CB1 and CB2 receptors are associated with major depression and bipolar disorder, while the CB1 knockout mice showed significant depression-like behaviors [9, 10]. Besides, the selective estrogen receptor modulators (SERMs), e.g., tamoxifen, are used to treat estrogen receptor (ER)-positive breast cancer and osteoporosis. Interestingly, as indicated as a potential ER-independent target, tamoxifen binds to cannabinoid receptors (CBRs) with affinity in the low concentration range and acts as an inverse agonist [11]. Suggesting that the estrogen receptor activation could promote CBRs to undergo the relevant responses.

Many of the health complications associated with menopause in women are directly related to decreased functions of the ovarian hormone, primarily 17β-Estradiol (E2) deficiency. Therefore, the importance of physiological hormone replacement therapies, mainly including E2 replacement therapy (ERT), has attracted increasing attention in the treatment of postmenopausal women [12].

Currently, hormone therapy in menopausal syndrome is still controversial. Firstly, in animal and cell experiments, the ERT has shown anti-inflammatory and antioxidant effects, stabilizing intracellular calcium levels, modulating the cholinergic system, and ultimately improving cognitive functions and depressive-like behaviors in ovariectomized (OVX) mice [13]. However, many clinical trials have revealed no significant changes in the incidence of the menopausal syndrome as well as the cognitive and depressive symptoms by ERT [14].

The selective activation of estrogen receptors (ERs) is probably the most important cause of unsuccessful brain protection and adverse effects in clinical treatment by E2 of physiological doses in the ERT. The cellular receptors of E2 are crucial mediators of E2 functions, including members in the nuclear receptor family (e.g., ERα and ERβ) and membrane ERs, e.g., G protein-coupled estrogen receptor (GPER) [15, 16]. The ERα promotes mammary and uterine maturation and maintains metabolic and skeletal homeostasis, while ERβ shows significant effects on the CNS. Generally, in comparison to ERβ, the ERα shows a higher functional activity that can be activated by physiological doses of E2 to suppress the brain-protective effect of ERβ [17].

Furthermore, studies in vitro have revealed no anti-inflammatory effects of E2 with physiological concentrations in the presence of activated microglia, while an increased dose of E2 may activate the suppressed ERβ [18–20]. Therefore, in this study, the OVX mice were treated with supraphysiologic doses of E2 (sE2) to evaluate their efficacy and potential toxicity on menopause-related neuropsychiatric symptoms.

## Materials and methods

### Animals

Female C57/BL6J mice (5 months old) were purchased from Weitong Lihua Experimental Animal Technology (Beijing, China) and carefully reared in the SPF laboratory of the animal experimental center of Shandong University. All mice were housed in a pathogen-free condition at 23 °C and 45% humidity with a photoperiod of 12-h light and 12-h dark and fed with sterile food and drinking water. After a week of acclimatization, the animals were randomly divided into three experimental groups, i.e., OVX, sham, and OVX + sE2 groups. All animal experiments followed the ARRIVE guidelines and were conducted in accordance with the U.K. Animals (Scientific Procedures) Act, 1986 and related guidelines. Completely randomized design and blinding were adopted in animal experiments.

### Ovariectomy

Mice were anesthetized with isoflurane (2.5%) after the lower back was shaved and cleaned with betadine and then an alcohol swab. A small incision was then made into the muscle wall with the uterine horn located and drawn through the muscle layer using blunt forceps. The ovaries were removed after the oviduct was ligated. The muscle layer was then sutured to close, while the skin was stapled. The sham-operated animals were treated with the same procedure without ligating the fallopian tube and the ovary excision. Finally, mice were placed in a heated cage until they recovered from anesthesia. Drug or control solvent injections were performed one week after surgery.

### Animal treatments

E2 was purchased from Med Chem Express (Beijing, China) and dissolved in olive oil. The mice in the OVX + sE2 group received a daily intraperitoneal injection of E2 (0.5 mg/kg) for 5 weeks. This dose of E2 was 2–3 times higher than that of the circulating serum levels in the normal maintenance of mice [21, 22]. For the preliminary, E2 serum concentrations were measured three days after E2 injection. The same volume of solvent was used in both OVX and sham-operated groups of mice. The body weights and food intakes in mice were recorded daily with the E2 dosage adjusted according to the change in body weights.

### Open field test

The open field test consisted of 5 min trial in a white opaque arena of 45 cm × 45 cm × 30 cm in size (Maze Engineers, Boston, USA). The test arena was divided equally into nine square quadrants. Each mouse was placed into the central quadrant of the open field and allowed to freely explore the arena. The trajectory, the total distance moved, and the time spent in the central area of each mouse was recorded by the camera and analyzed using ANY-mazeTM video-tracking software (Stoelting Co., Chicago, IL, USA).

### Y-maze test

The Y-maze test was carried out based on the method previously described [23]. Briefly, each mouse was placed in the arm designated (A) of the Y-maze and allowed to freely explore the maze for 5 min. The alternation score (%) was calculated as [(the number of three successive arm choices that include one of each arm) / (total arm entry − 2)] × 100. An arm entry was counted when all four limbs of the mouse were within an arm.

### Forced swim test

A forced swim test was performed according to the method previously described [24]. Briefly, the mice were placed into plastic buckets, each 18 cm in diameter and 25 cm in height and filled with water, and maintained at 23 °C. After 1 min of habituation, the immobility time (in the unit o sec) of the mice were recorded for a period of 5 min.

### Sucrose preference test

Mice were habituated to 1% sucrose water for 3 days prior to the test. After 24 h deprivation of water and food, mice were provided with 1% sucrose water and pure water. The consumption levels were measured after 24 h testing. Sucrose preference (%) = (sucrose water intake/(sucrose water intake + pure water intake)) × 100.

### Tissue processing

After the behavioral tests, the mice were deeply anesthetized with diethyl ether (10%, Buxco Electronics, Inc., Wilmington, NC, USA) and sacrificed for the preparation of blood extraction and brain tissue collection. Body, and brainweights were recorded. The cerebral index was expressed as: fresh brain weight/body weight. One part of the brain tissue of each mouse was fixed in 4% paraformaldehyde for subsequent histopathological analysis. After the brain tissue was dehydrated, embedded in paraffin, and sectioned with 6 μm thickness. The other part of the brain tissue was frozen in liquid nitrogen for subsequent RNA extraction, proteomic analysis and untargeted metabolomic analysis.

### Hormone assay

The serum level of E2 was measured with ELISA immunoassay (Estradiol EIA kit, Oxford Biomedical Research, Oxford, MI) according to the manufacturer’s instructions.

### Immunostaining

The immunohistochemical experiments were conducted as previously described [25]. The antibodies used in this study included an anti-Iba1 antibody (1:50; ab178846; ab283319; Abcam, MA, USA), an anti-CD86 antibody (1:200; ab220188; Abcam, MA, USA), anti-IL-1β antibody (1:200; ab254360; Abcam, MA, USA), anti-TNF-α antibody (1:150; ab1793; Abcam, MA, USA), anti-IL-6 antibody (1:200; ab290735; Abcam, MA, USA), anti-ERα antibody (1:200; ab32063; Abcam, MA, USA), anti-GPER antibody (1:200; PA5-77396; Invitrogen, Karlsruhe, Germany), anti-ERβ antibody (1:150; PA1-311; Thermo Fisher Scientific, Shanghai, China), anti-NF-κB p65 antibody (1:200; ab32536; Abcam, MA, USA), anti-COX1 antibody (1:500; ab109025; Abcam, MA, USA), anti-CB1 antibody (1:200; ab3558; Abcam, MA, USA), anti-IL-1 (1:200; ab254360; Abcam, MA, USA), Alexa Fluor 488 secondary antibody (1:1,000; Invitrogen, Karlsruhe, Germany), Alexa Fluor 594 secondary antibody (1:1,000; Invitrogen), and IgG H&L (HRP) antibody (1:500; ab97051; Abcam, MA, USA). The nuclei were stained with DAPI (Sigma-Aldrich, Beijing, China) and images were captured on the LSM700 laser scanning confocal microscope (Zeiss, Tokyo, Japan) with the mean fluorescence intensities (MFI) determined using the FlowJo software [26].

### Proteomic analysis

Proteomic analysis (OVX and OVX + sE2 groups, each n = 5) was performed using mass spectrometry in data‐dependent acquisition mode. To designate significant changes in protein expression, a fold-change of > 1.2 or < 0.83 and a P-value of < 0.05 using Student’s t-test were set as cut-off values. The differential proteins were mapped to the KEGG database (https://www.kegg.jp/kegg/pathway.html) to enrich KEGG pathways.

### Untargeted metabolomic analysis

Untargeted metabolomics analysis of both OVX and OVX + sE2 groups (each n = 5) was performed by LC–MS/MS as previously described [27]. The LC–MS/MS analysis was conducted on a quadrupole electrostatic field orbitrap high-resolution mass spectrometer (Thermo Fisher Scientific, USA). Assignment of metabolites was determined based on the published data and publicly available databases such as HMDB (http://redpoll.pharmacy.ualberta.ca/hmdb/HMDB/), KEGG (http://www.genome.jp/kegg), PubChem compound database (http://pubchem.ncbi.nlm.nih.gov), and SMPDB (http://smpdb.ca).

### Hematoxylin and eosin (HE) staining

HE staining was conducted according to the routine protocols. Briefly, the brain sections of mice were deparaffinized, rehydrated, stained with hematoxylin (Biyuntian, Beijing, China), differentiated with acidic ethanol, stained with eosin (Biyuntian, Beijing, China), dehydrated, and mounted with Permount (Fisher Scientific, Shanghai, China).

### Nissl staining

Nissl staining was performed to detect neuronal injury. The brain sections of mice were stained with 1% Cresyl Violet (C5042, Sigma-Aldrich, St Louis, MO, USA) and covered with 50% glycerin. The stained sections were photographed by a Keyence microscope (BZ9000, Osaka, Japan).

### Cell lines and treatments

The microglial cell line BV-2 was purchased from the Cell Bank of the Chinese Academy of Sciences (Shanghai, China) and was cultured in a matched dedicated complete medium (CM-0493; Procell, Wuhan, China) at 37 °C with 5% CO_2_. The hippocampal cell line HT22 was purchased from the Procell Cell Bank (Wuhan, China) and was cultured in Dulbecco’s Modified Eagle Medium (DMEM) supplemented with 10% fetal bovine serum (Pan-Biotech, Aidenbach, Germany).

Primary microglia and neurons were extracted from the brains of neonatal female C57/BL6J mice (n = 32; 1–2 days old) as previously described [28]. The microglia were cultured in Neurobasal-A medium containing 2% B27, 2-mM L-glutamine, 50-U/ml penicillin, and 50-U/ml streptomycin (Gibco, USA). The culture medium for the neuron was DMEM/F12 containing 10% fetal bovine serum, 1 mM sodium pyruvate, 2 mM l-glutamine, 100 mM nonessential amino acids, 50 U/ml penicillin, and 50 mg/ml streptomycin (Gibco, USA).

BV-2 cells or primary microglia were seeded in the 24-well culture plates with cell-climbing slices. Then, the cells were exposed to E2 of different concentrations (0–3200 nM) for 24 h. To investigate the effect of E2 on the activated microglia, the cells were exposed to lipopolysaccharide (LPS; 100 ng/ml, Sigma) for 1 h and then co-treated with both LPS and E2 at either a high (800 nM) or a low concentration (200 nM). Inhibition of NF-κB was performed using the NF-κB inhibitor QNZ (EVP4593, Med Chem Express, Beijing, China) at a concentration of 30 nM for 24 h.

Both BV-2 and HT22 cells or primary microglia and neurons were co-cultured in a 2-layered co-culture chamber with the HT22 cells or primary neurons (2 × 10^5^ cells) cultured in the upper layer and BV2 cells or primary microglia (2 × 10^5^ cells) cultured in the lower layer. The intermediate membrane allowed the passage of culture fluid but not cells. Following the establishment of the co-culture system, cells were incubated with E2 (0 or 800 nM) for 0 to 72 h, respectively. Then, the viability of HT22 cells or primary neurons was determined using the CCK-8 assay.

### CCK-8 assay

The cell counting kit-8 (CCK-8) assay kit (Dojindo, Shanghai, China) was used to evaluate the effects of E2 at different concentrations and treatment durations on cell viability according to the manufacturer’s protocol.

### RNA extraction and qPCR

Total RNA was extracted from the brain tissues of mice or cells after the treatments using RNA Extraction Kit (Qiagen Sciences, Maryland, USA) according to the manufacturer's protocol. RNA was reverse transcribed via the Super Script II Reverse Transcriptase Kit (Invitrogen, Beijing, China) followed by quantitative real-time PCR (qRT-PCR) using Power SYBR Green PCR Master Mix (Applied Biosystems, Foster City, USA) based on primers given in Additional file 4: Table S1.

### Suppression of estrogen receptors using siRNA

ERα siRNA, ERβ siRNA, GPER siRNA, and control siRNA were purchased from Thermo Fisher Scientific (Shanghai, China). The transfections were performed as previously described [29]. The siRNA sequences were given in Additional file 4: Table S2.

### Western blot analysis

Western blot analysis was performed as previously described [30]. The antibodies used in this study included an anti-CD86 antibody (1:200; ab220188; Abcam, MA, USA), anti-NF-κB p65 antibody (1:200; ab32536; Abcam, MA, USA), anti-GADPH antibody (Santa Cruz Biotechnology, CA, USA), anti-Histone H3 (1:1000; ab1791; Abcam, MA, USA), and goat anti-Rabbit IgG H&L (HRP; 1:2000; ab6721; Abcam, MA, USA). The original image of the WB stain is given in Additional file 5.

### Data analysis and statistics

Statistical analyses were performed with the SPSS software (SPSS Standard version 24.0, SPSS). The statistical analysis among groups was performed using one-way ANOVA followed by Tukey’s post hoc test. The significance of differences between the two groups was determined by Student’s t-test (unpaired, two-tailed). The qRT-PCR quantification was conducted using the 2^−ΔΔCt^ method. A value of P < 0.05 was considered statistically significant.

## Results

### sE2 aggravates depressive-like behaviors in OVX mice

**Fig. 1:**
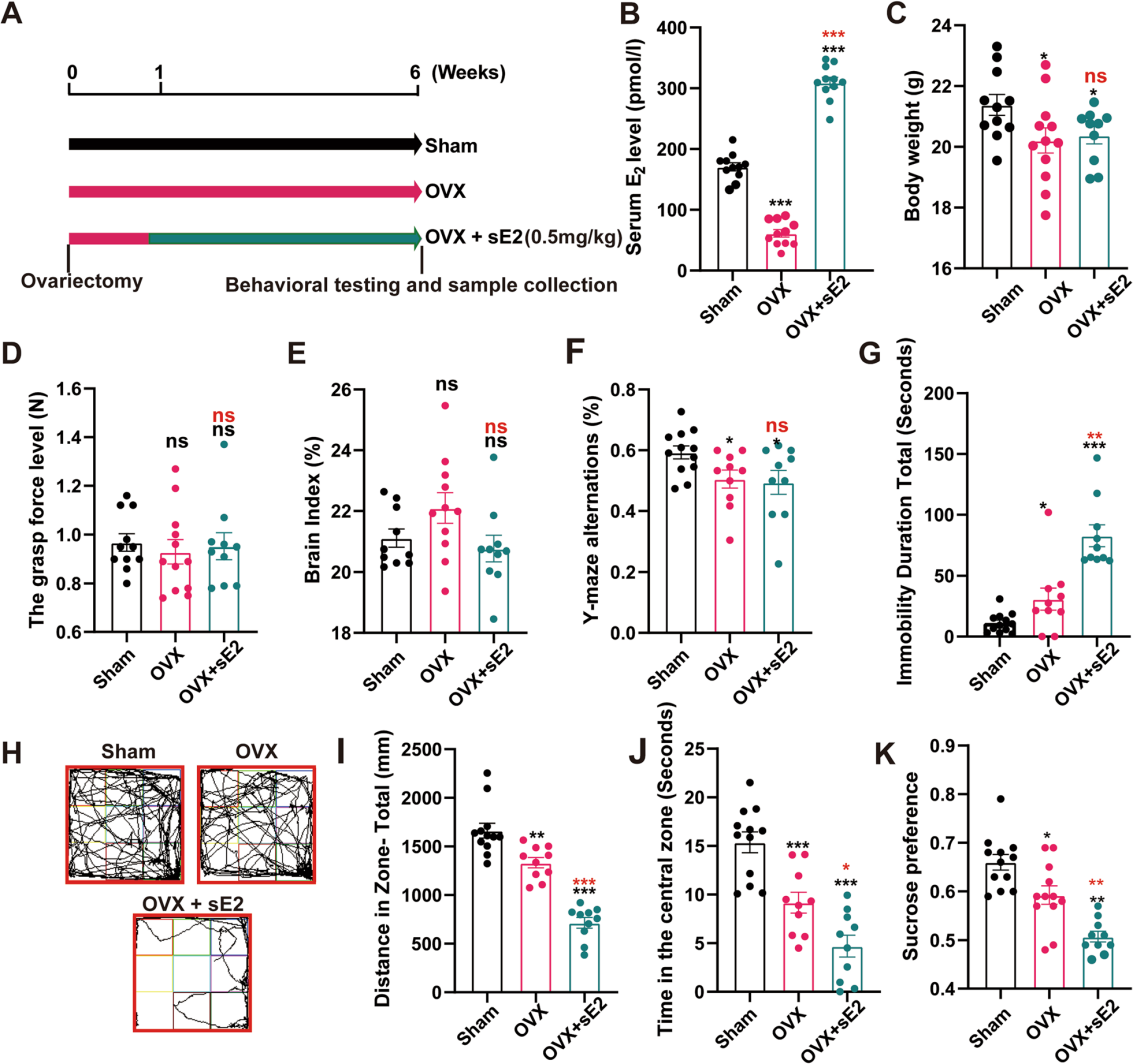
Supraphysiological doses of 17β-Estradiol (sE2) exacerbate depression-like behaviors in ovariectomized mice. **A** Schematic illustration of the workflow of animal experiments. **B** Serum 17β-Estradiol levels in all groups of mice. **C–E** Body weight, grasp, and brain index in the different groups of mice before sacrifice. **F** Alternation score in the Y maze task. **G** The immobility time of mice in the forced swim test. Representative motion track (**H**), distance (**I**), and time spent in the center quadrant (**J**) of the mice in the open field. The sucrose preference index (**K**) was measured using sucrose preference test. Students t-test is performed to determine the significant difference based on P < 0.05 (*), P < 0.01 (**), and P < 0.001 (***), respectively, in comparison to the sham group (as indicated by black asterisks and “ns”) or the OVX group (as indicated by red asterisks). ns: not significant difference. Data are presented as mean ± the standard deviation (SD) of at least ten animals per group

We selected an E2 dose of 0.5 mg/kg for these studies based on the results of our pre-experiments to increase the level of E2 in the peripheral blood compared with the physiological levels in OVX mice (Additional file 1: Figure S1). The OVX mice were used as the menopause models to determine the therapeutic effect of sE2 on menopausal neurodegenerative pathology. The mice were treated with sE2 one week after surgery (Fig. 1A). We found that after 5 weeks of sE2 treatment, the peripheral blood E2 concentration of mice increased about twofold compared with the physiological concentration of sham-operated mice (Fig. 1B). The results showed that the sE2-treated mice showed no significant changes in both body weight and grasp force compared to the OVX group, with largely identical body conditions in both groups of mice (Fig. 1C, D). No significant changes were observed in brain weight index in all groups of mice (P > 0.05; Fig. 1E).

The Y-maze test was performed to assess the working memory in mice. The spontaneous alternating response rate of the mice in the OVX group was significantly decreased compared to that of the sham group (P < 0.05), whereas the addition of sE2 caused insignificant changes in the capacity of working memory in OVX mice (Fig. 1F).

Both the forced swim test, open field test and sucrose preference test were performed to evaluate depression-like behaviors in mice. The results of the forced swim test showed that the immobility time of mice in the OVX group was significantly prolonged compared with that of the sham group, with the increasing trend further aggravated by the sE2 treatment (P < 0.01; Fig. 1G). Similarly, the results of the open field test showed that the treatment of sE2 further reduced the distance traveled and the time spent in the central quadrant of the open field by the OVX mice (Fig. 1H–J). Meanwhile, the results of sucrose preference test showed that sE2-treated mice had a reduced preference for sucrose water (Fig. 1K). These results suggested that sE2 aggravated depression-like behaviors in OVX mice without affecting their short-term memory. To further explore the mechanism underlying the functions of sE2, we subsequently performed the histological analysis of the brain tissues of mice.

### sE2 aggravates microglial response and neuroinflammation in the hippocampus and cortex of OVX mice

**Fig. 2:**
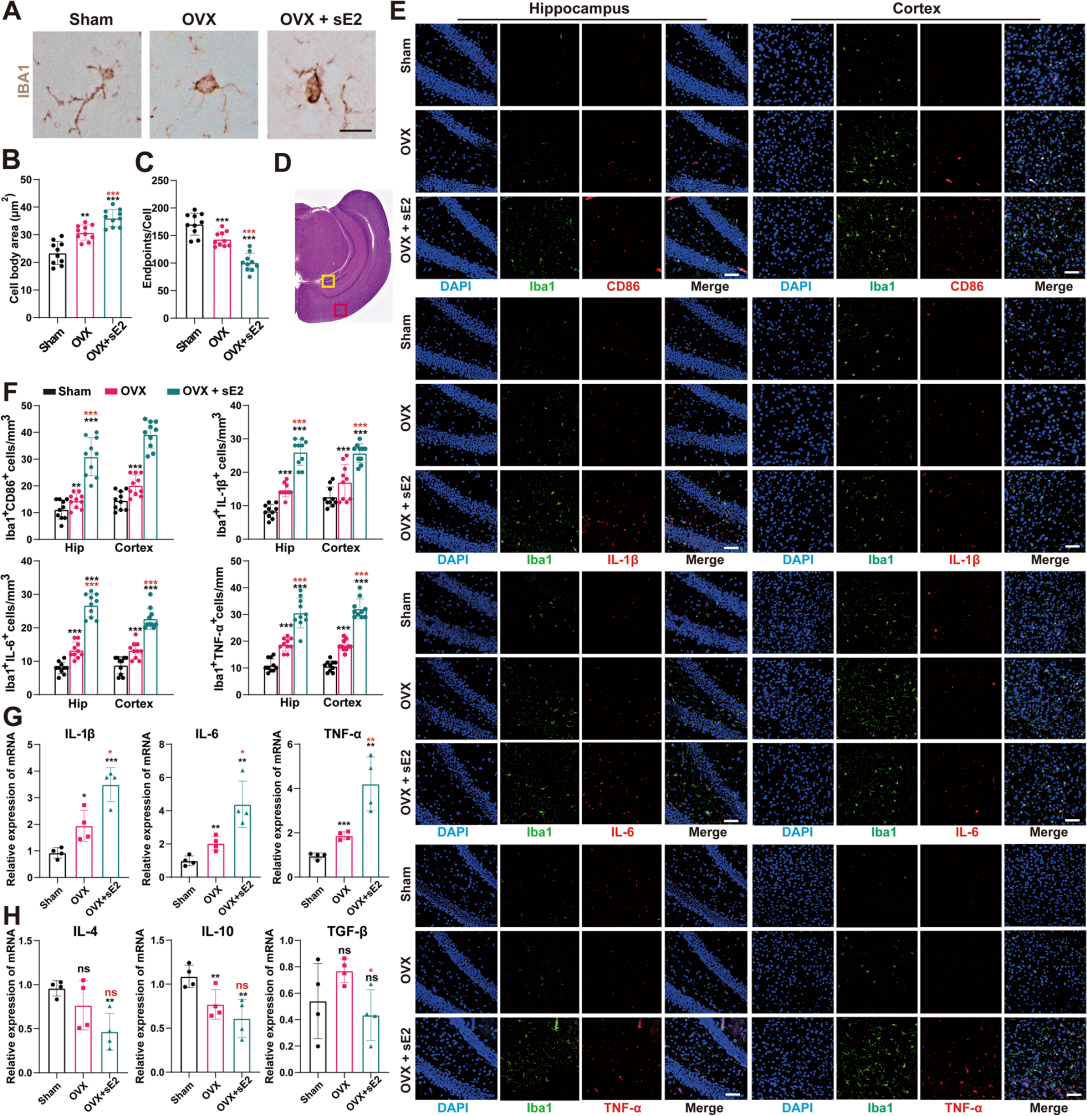
Supraphysiological doses of 17β-Estradiol (sE2) exacerbate microglial response and neuroinflammation in the brains of ovariectomized mice. Immunohistochemical staining is used to label microglia in paraffin sections of mouse brain tissues with an anti-Iba1 antibody. Representative pictures (**A**) and quantification of the cell body area and endpoints of Iba1-labeled microglia in each group of mice (**B**, **C**). Bar = 5 µm. **D** Areas for immunofluorescence analysis marked on HE-stained coronal brain sections. The hippocampus is marked in yellow squares and the cortex is marked in red squares. **E** Typical images of microglia (Iba1-labeled) and M1 polarization markers (i.e., CD86, IL-1β, IL-6, and TNF-α) co-located by immunofluorescence. Bar = 50 µm. **F** Quantification of the number of positive cells for microglia M1 polarization markers per field in each group of mice. The mRNA levels of proinflammatory factors IL-1β, IL-6, and TNF-α (**G**), and anti-inflammatory factors IL-4, IL-10, and TGF-β (**H**) are evaluated by qPCR analysis. Student’s t-test is performed to determine the significant difference based on P < 0.05 (*), P < 0.01 (**), and P < 0.001 (***), respectively, in comparison to the sham group (as indicated by black asterisks and “ns”) or the OVX group (as indicated by red asterisks). ns: no statistical significance. Data are presented as mean ± the standard deviation (SD) of at least four animals per group

Here, we focused on slices made at the ventral hippocampal location because the ventral hippocampus is mainly associated with anxiety-like behaviors [31]. Iba1 is a commonly used pan-marker of microglia and macrophages. Morphological changes (i.e., hypertrophy and the number of branch endpoints per cell) in Iba1-positive cells are generally considered indicators of microglial activation [32]. The immunofluorescent staining of Iba1 was performed to determine the effects of sE2 on the reactive microglia, followed by microscopic and morphological analyses (Fig. 2A). The results showed that sE2 significantly aggravated the OVX-induced elevation in microglial cell body areas and the number of branch endpoints (P < 0.001; Fig. 2B, C).

To investigate the role of sE2 in regulating the microglial response and neuroinflammation following the depressive-like behaviors in mice, we evaluated the levels of the biochemical markers for the microglial response, including cell surface marker CD86 and proinflammatory cytokines IL-1β, IL-6, and TNF-ɑ, in cortical and hippocampus regions by immunofluorescence (Fig. 2D, E). The results showed that compared with the sham group, the number of microglia with proinflammatory phenotype in the cerebral cortex and hippocampus of mice in the OVX group was significantly increased, with the increasing trend further aggravated by the sE2 treatment (P < 0.001; Fig. 2F).

Then, the qRT-PCR analysis was performed to evaluate the gene expressions of proinflammatory (IL-1β, IL-6, and TNF-ɑ) and anti-inflammatory cytokines (IL-4, IL-10, and TGF-β) in the brain tissues of mice. The results showed that sE2 treatment significantly increased the expression of proinflammatory cytokines and inhibited anti-inflammatory cytokines in the brain tissues of OVX mice (Fig. 2G, H). Next, we performed the in vitro experiments to further confirm these findings.

### High concentrations of E2 promote the pro-inflammatory response state of microglia

**Fig. 3:**
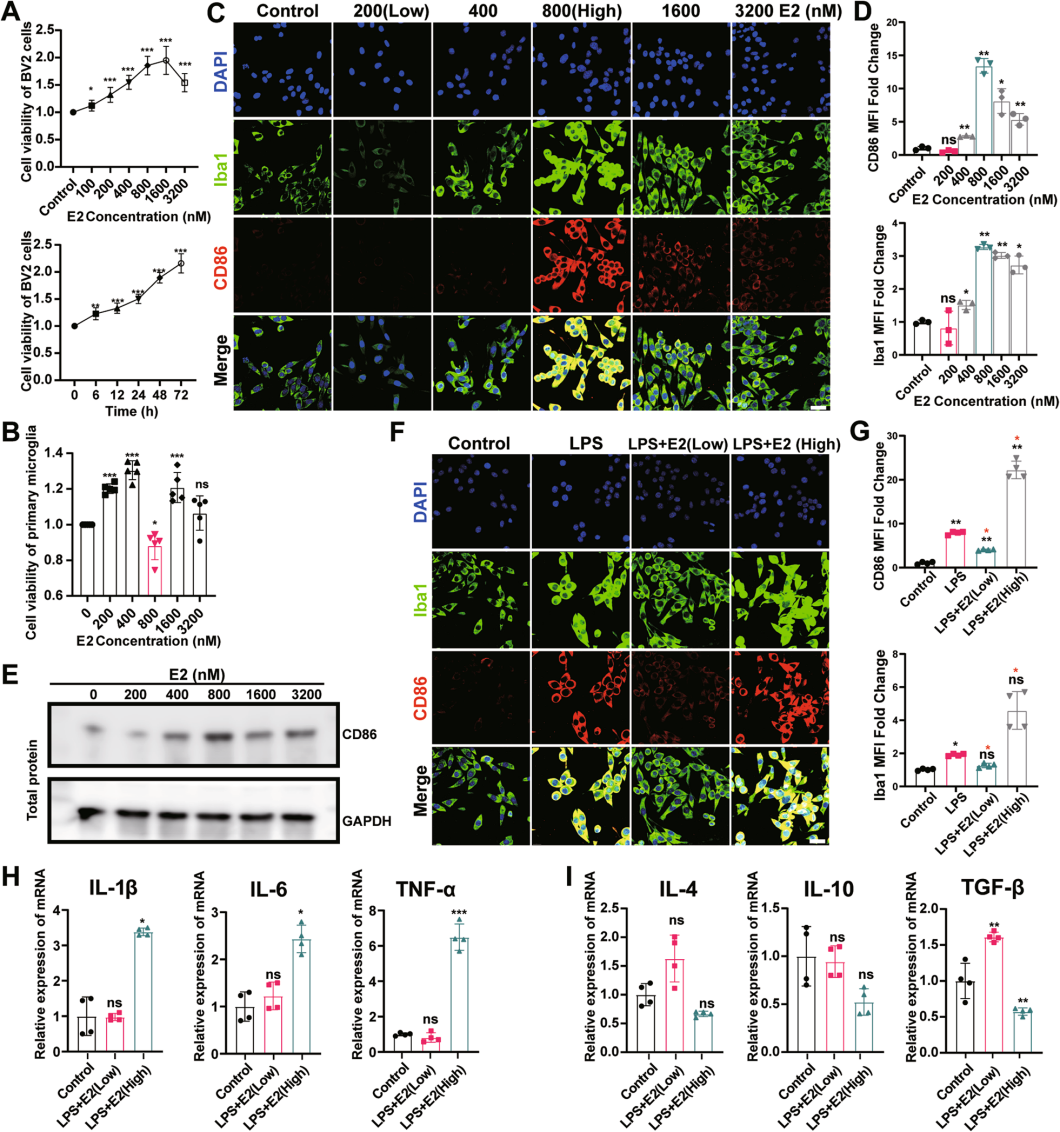
High concentrations of 17β-Estradiol (hE2) promote microglial microglial response.** A** Viability of BV-2 cells after treatment with E2 for various durations and concentrations. **B** Viability of primary microglia after treatment with E2 for various concentrations. BV2 cells are fixed and immunostained for Iba1 (green) and CD86 (red) (**C, F**). Bar = 30 µm. Cells are incubated with E2 of 0 nmol/L to 3200 nmol/L for 24 h (**C, E**) or pre‐treated with 100 ng/mL of LPS for 1 h and then co‐treated with LPS and E2 (200 or 800 nmol/L) for 24 h (**F**). The mean fluorescence intensity of CD86 or Iba1 and the levels of Iba1 intensity expressed as a relative change in comparison with untreated cells (**D, G**). E Representative band of protein expression. The mRNA levels of proinflammatory factors IL-1β, IL-6, and TNF-α (**H**), and anti-inflammatory factors IL-4, IL-10, and TGF-β (i) are evaluated by qPCR. Students t-test is performed to determine the significant difference based on P < 0.05 (*), P < 0.01 (**), and P < 0.001 (***), respectively, in comparison to the control group as indicated by black asterisks or ns and the LPS + E2 (Low) group as indicated by red asterisks. ns: no statistical significance. Data are presented as mean ± the standard deviation (SD). Each experiment is repeated independently in three

To evaluate the effect of E2 on the survival of BV2 cells, the CCK-8 assays were performed to evaluate the cell viability. The results revealed no significant change in the cell activity of BV2 by the treatment of E2 with a wide range of concentrations (100–3200 nM; Fig. 3A). Moreover, the cell activity of BV2 was increased with the extension of the treatment time of E2 (400 nM; Fig. 3A). However, in primary microglia, E2 failed to increase cell viability at a concentration of 800 or 3200 (nM/L; Fig. 3B).

Immunofluorescence was used to further analyze the proinflammatory status markers of microglia, namely Iba1, and CD86. The results revealed no effect of low-dose E2 (100 nM) on the expression of Iba1 and CD86, which was significantly activated by high-dose E2 (hE2; 800 nM) (Fig. 3C, D) in a dose-dependent manner. The above results were replicated in primary cultured microglia (Fig. 3E). Therefore, the concentration of 800 nM/L of E2 was selected in the subsequent in vitro experiments.

Because LPS is a prototypical inducer of mouse microglial activation [38], the effects of E2 on the microglial response to LPS stimulation were further evaluated. The results showed that low concentrations of E2 significantly inhibited the LPS-induced BV2 cell proinflammatory response state, which was significantly promoted by hE2 (Fig. 3F, G). These results were consistent with those derived from the qPCR analysis, showing that low concentrations of E2 inhibited the elevation of LPS-induced proinflammatory cytokines and the decrease of anti-inflammatory cytokines, whereas hE2 was revealed with the opposite effect (Fig. 3H, I).

### hE2 causes abnormal responses of microglia via ERα/NF-κB pathway

**Fig. 4:**
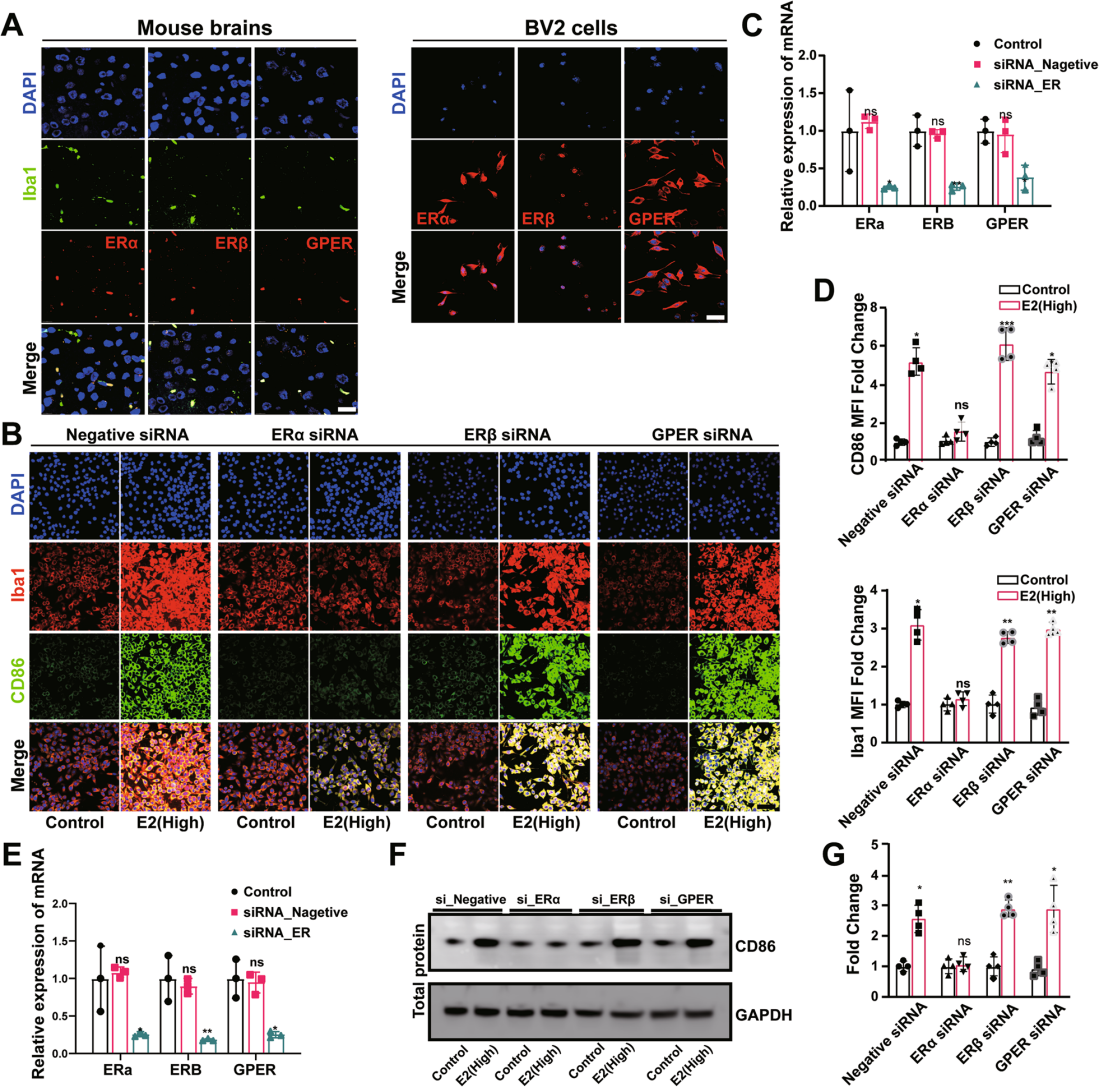
Microglial response caused by high-dose of estrogen (hE2) via ERα. **A** Representative images of microglia (Iba1-labeled) and estrogen receptors (Erα, ERβ, and GPER) co-located in mouse brain and BV2 cells by immunofluorescence. Bar = 15 μm. The siRNAs are used to suppress different estrogen receptors (ERs) in BV2 cells. Negative siRNA is used as the negative control. The effects of hE2 on CD86 and Iba1 are subsequently analyzed. **B** Representative images of microglia (Iba1-labeled) and M1 polarization markers (CD86) co-located by immunofluorescence. **C** qPCR analysis of ER proteins in BV2 cells transfected with different siRNAs to suppress the corresponding ER. **D** Quantification of the mean fluorescence intensity per cell with the indicated number of cells. **E** The siRNAs are used to suppress different estrogen receptors (ERs) in primary microglia. Results of qPCR analysis of ER proteins in primary microglia transfected with different siRNAs to suppress the corresponding ER. Representative band (**F**) and quantification (**G**) of protein expression in primary microglia. Students t-test is performed to determine the significant difference based on P < 0.05 (*), P < 0.01 (**), and P < 0.001 (***), respectively, in comparison to the control group as indicated by black asterisks or “ns” (i.e., no statistical significance). Data are presented as mean ± standard deviation (SD)

Studies have shown that E2 modulates its effects at cellular levels via its receptors [33]. To confirm the localization of ER in microglia, we conducted immunofluorescence experiments to show that ERα, Erβ, and GPER were present in the microglia of both mouse brain and BV2 cells, with ERβ mainly localized in the nucleus and CPER mainly outside the nucleus, while ERα was widely expressed in BV2 cells (Fig. 4A).

A group of siRNAs was used to silence the corresponding ERs to identify the specific type of ERs that participated in the process of the hE2-mediated proinflammatory response of microglial regulation. The results intriguingly showed that when the ERα was silenced, the effects of hE2 on both CD86 expression and Iba1 were interrupted (Fig. 4B–G). These results suggested that hE2 bound to ERα activated microglia to promote their abnormal responses both in BV2 cells and primary microglia.

**Fig. 5:**
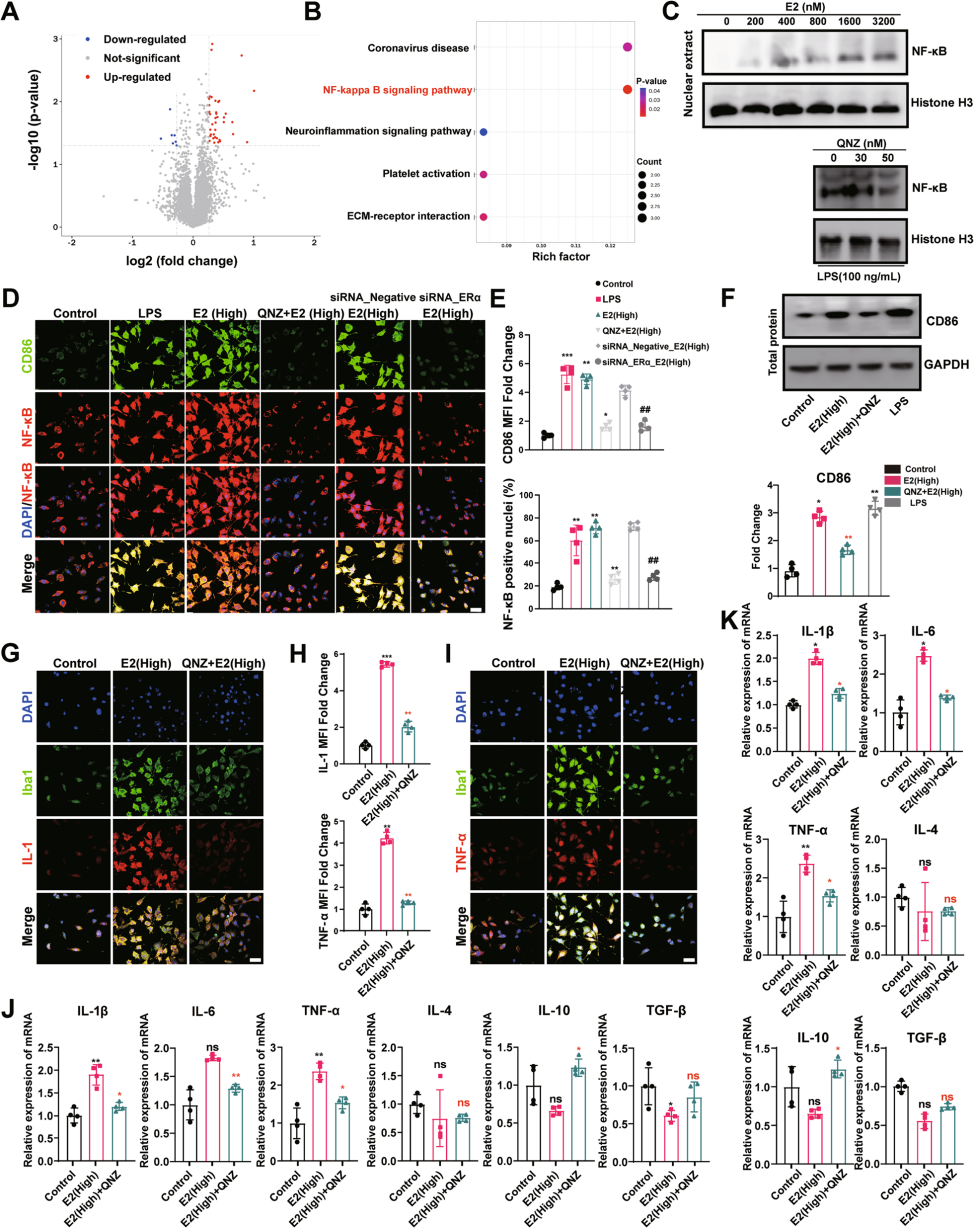
High concentrations of 17β-Estradiol (hE2) promote microglial response through the NF-κB signaling pathway. **A** Volcano plot of protein abundances changes in OVX vs. OVX + sE2 mice brians, based on proteomics analysis. **B** KEGG pathway enrichment analysis of differentially expressed proteins. QNZ is used as an inhibitor of NF-κB activation. The effects of hE2 on CD86 protein expression levels and NF-κB activation are analyzed. **C** Western blot analysis was used to examine the effects of E2 or QNZ of different concentrations on NF-κB activation in primary microglia. **D** Typical images of microglia M1 polarization markers (CD86 labeled) and NF-κB signaling pathway (p65) co-located in BV2 cells by immunofluorescence. Bar = 15 μm.** E** The relative quantification of CD86 and NF-κB-stained positive cells. Nuclear translocation of NF-κB is determined by immunofluorescence microscopy. **F** Representative band (up) and quantification (down) of protein expression in primary microglia. Typical images of microglia (Iba1-labeled) and NF-κB signaling pathway (IL-1 and TNFα) co-located in BV2 cells by immunofluorescence (**G, I**). Bar = 15 μm. **H** Quantification of the mean fluorescence intensity per cell with the indicated number of cells. The mRNA levels of proinflammatory factors IL-1β, IL-6, and TNF-α, and anti-inflammatory factors IL-4, IL-10, and TGF-β are evaluated by qPCR in primary microglia (**K**) or BV2 cells (**J**). Student’s t-test is performed to determine the significant difference based on P < 0.05 (*), P < 0.01 (**), and P < 0.001 (***), respectively, in comparison to the control group as indicated by black asterisks or “ns,” to the E2 (High) group as indicated by red asterisks, and to the siRNA negative group as indicated by symbol “#.” ns: no statistical significance. Data are presented as mean ± the standard deviation (SD). Each experiment is repeated independently three

Subsequently, the results of proteomic analysis showed that NF-κB pathway signaling was significantly activated in the brain of sE2-treated mice compared with the OVX group (Fig. 5A and B). As in vitro results, the NF-κB expression in the nucleus of primary microglia was increased with elevated concentration of E2 (Fig. 5C), while both LPS and hE2 caused M1 polarization of microglia and nuclear translocation of NF-κB, which in turn were inhibited by NF-κB inhibitor QNZ (Fig. 5D–F). Meanwhile, ERα-siRNA inhibited hE2-induced NF-κB activation and CD86 expression (Fig. 5D, E).

To further determine the correlation between NF-κB activation and microglial activation, immunofluorescence staining was performed for two additional markers (i.e., IL-1 and TNF-α) of the NF-κB pathway and Iba1. The results showed that hE2 promoted the expressions of IL-1 and TNF-a while activating microglia with these effects inhibited by QNZ (Fig. 5G–I). These results were consistent with those derived from the qPCR analysis, showing that QNZ inhibited the elevation of hE2-induced proinflammatory cytokines and the decrease of anti-inflammatory cytokines in both BV2 cells (Fig. 5J) and primary microglia (Fig. 5K).

To summarize, these results suggested that hE2 activated microglia by activating the ERα/NF-κB pathway to promote the pro-inflammatory response of microglia.

### sE2/hE2 causes neuronal damage by regulating the response of microglia

**Fig. 6:**
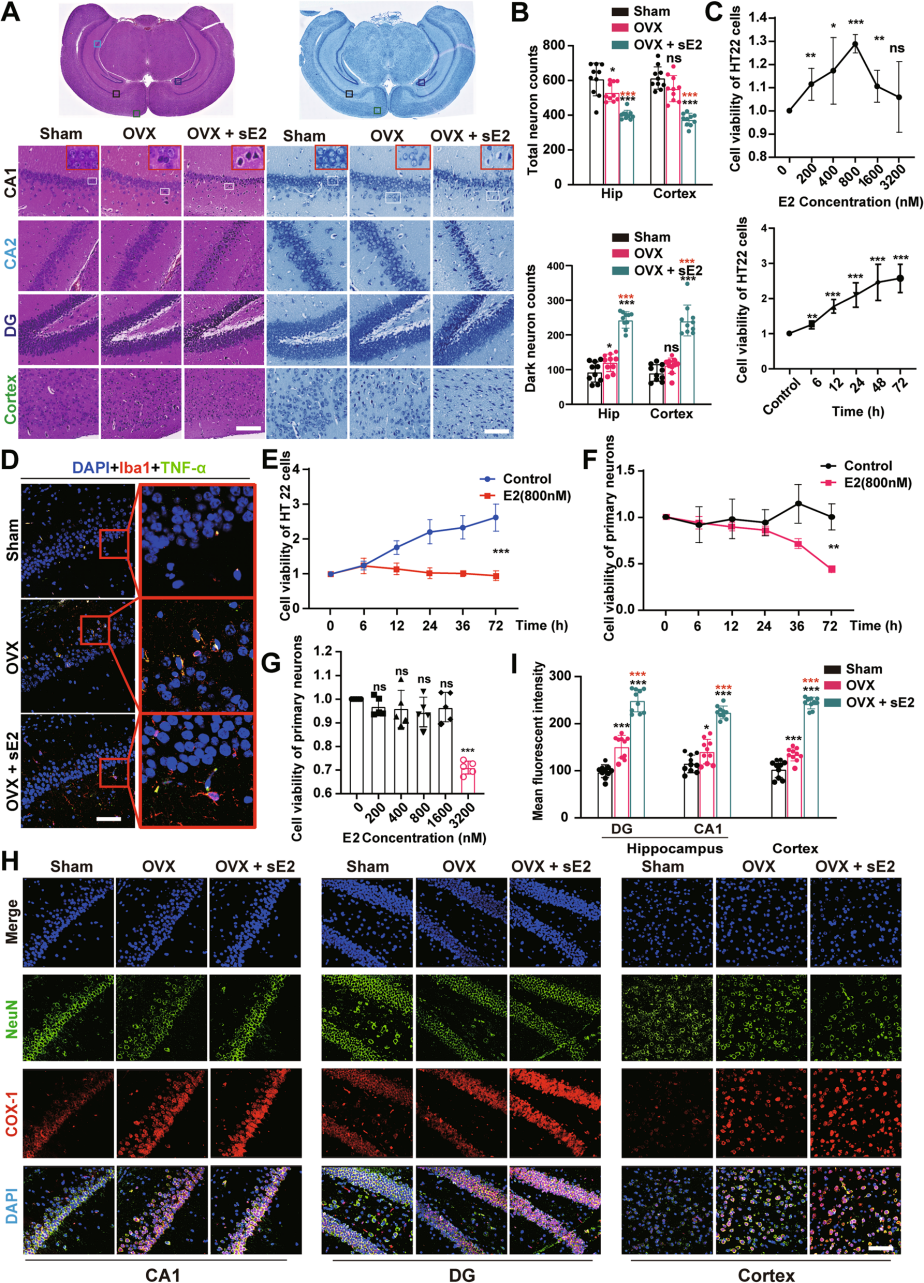
Supraphysiological doses of 17β-Estradiol (sE2) exacerbate neuronal damage in ovariectomized mice. Representative images of HE (left) and Nissl (right) staining of coronal brain sections. Bar = 50 µm. Black, light blue, and dark blue boxes indicate the hippocampal CA1, CA2, and DG areas; green boxes indicate the cortex. **B** Quantification of total neuron (up) and cell death by Nissl staining in neurons (down). **C** Viability of HT22 cells treated with E2 for various treatment durations and concentrations. **D** Typical images of microglia (Iba1-labeled) and M1 polarization markers (TNF-α) co-located by immunofluorescence. Cytotoxicity is detected by CCK-8 assays. HT22 cells (**E**) or primary neurons (**F**) are cultured either in E2 alone or co-cultured with microglia for 0–72 h. **G** Viability of primary neurons treated with E2 of various concentrations. **H** Typical images of neurons (NeuN-labeled) and COX1 co-located by immunofluorescence. Bar = 50 µm. **I** Quantification of the mean fluorescence intensity of COX1 per NeuN^+^ cell for (**H**). Student’s t-test is performed to determine the significant difference based on P < 0.05 (*), P < 0.01 (**), and P < 0.001 (***), respectively, in comparison to the sham or control groups as indicated by black asterisks or “ns” and to the OVX group as indicated by red asterisks. ns: no significant difference. Data are presented as mean ± the standard deviation (SD). Each experiment is repeated independently three. Data are based on a minimum of 10 animals in each group

The effects of E2 on the neurons in mice were further evaluated. The observations of the Nissl- and HE-stained brain tissue sections of mice revealed that the neurons in the hippocampus and cortex in the sham group were distinctly clustered together, with evident contour and transparent cytoplasm (Fig. 6A). In contrast, the nucleoli in the OVX group were pyknotic, showing the disordered arrangement of neurons. This type of damage was much worse in the sE2-treated mice, as shown by the quantitative data (Fig. 6B).

Subsequently, the effects of E2 on cytotoxicity and cell survival were evaluated by CCK-8 assays. The results unexpectedly showed no significant decrease in cell viability of HT22 cells treated with E2 of various concentrations and treatment durations (Fig. 6C). Compared with the OVX group, the sE2-treated mice showed significant microglial infiltration around the hippocampal neurons (Fig. 6D). It was speculated that the sE2 probably damaged neurons through activated microglia. This speculation was verified by co-culturing both HT22 and BV2 cells in the presence of hE2 for 72 h. The results showed that the cell viability of HT22 cells was significantly decreased under the influence of an hE2-stimulated BV2 cell medium (Fig. 6E). Similar results were also obtained in primary cells (Fig. 6F).

### sE2 induces brain metabolic disorders in ovariectomized mice

**Fig. 7:**
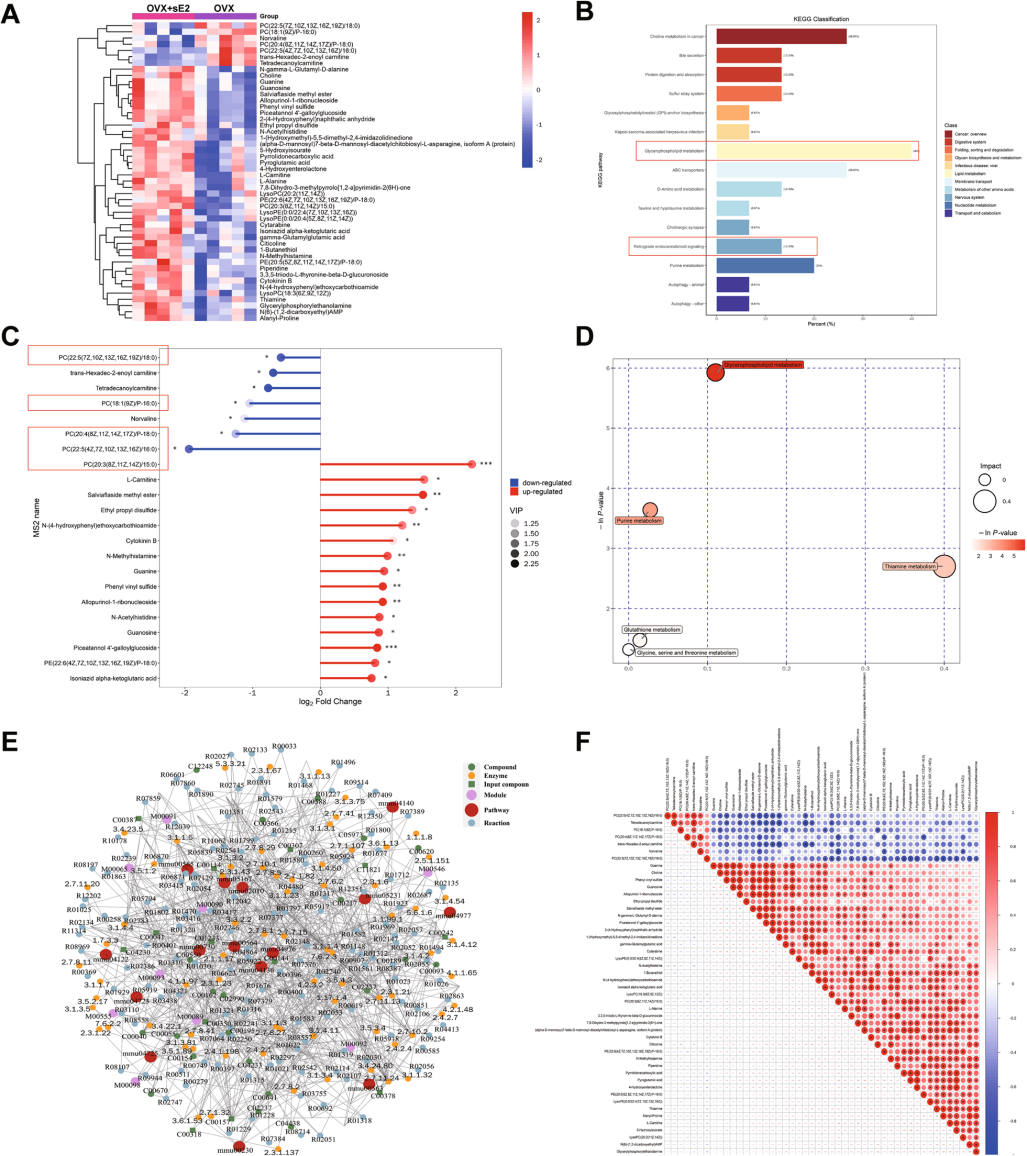
Analysis of untargeted metabolomics of brain from sE2‐treated OVX mice compared to vehicle‐treated OVX mice under positive ions.** A** Heatmap of 48 significantly changed metabolites based on untargeted metabolomics. **B** The KEGG enrichment analysis of the differential metabolites. The X-axis indicates the number of annotated metabolites under a given pathway as a percentage of all annotated metabolites. **C** Matchstick analysis of the differential metabolites. Red boxes are labeled for metabolites related to glycerophospholipid metabolism and retrograde endocannabinoid signaling. ANOVA is performed to determine the significant difference based on P < 0.05 (*), P < 0.01 (**), and P < 0.001 (***), respectively. **D** Pathway enrichment of differential metabolites. **E** Network analysis of the differential metabolites. **F** Heatmap of correlation analysis for differential metabolites. Each group contained 5 animals

Unlike HT22 cells, the high doses of E2 (3200 nM/L) inhibited cellular activity in primary neuronal cells (Fig. 6G). Considering the effects of menopause on brain metabolism in women, immunofluorescence co-localization staining of mouse neuronal cells (NeuN marker) and mitochondrial complex IV subunits (COX-1) was performed (Fig. 6H). The results showed that the sE2 significantly aggravated COX1 expression in the hippocampus and cortex of OVX mice brains (Fig. 6I; P < 0.001), suggesting that sE2 could be involved in the brain substance metabolism in OVX mice [34]. We then performed untargeted metabolomics analysis on the brains of OVX and OVX + sE2 mice (Fig. 7). After metabolite extraction, samples were processed in both positive and negative ion modes and analyzed separately.

In the positive ion mode, compared with the OVX group, a total of 48 differential metabolites (7 down-regulated and 41 up-regulated) were identified in the OVX + sE2 group (Fig. 7A). The KEGG enrichment analysis showed that sE2 affected metabolic pathways of glycerophospholipid metabolism, cholinergic synapse, retrograde endocannabinoid signaling, etc. (Fig. 7B). Meanwhile, a variety of metabolites related to both glycerophospholipid metabolism and retrograde endocannabinoid signaling were down-regulated (Fig. 7C, marked in red boxes), with glycerophospholipid metabolism showing the highest enrichment (Fig. 7D). Similarly, the results of regulatory network analysis and correlation analysis of differential metabolism also indicated that both glycerophospholipid metabolism and retrograde endocannabinoid signaling were the key metabolic pathways (Fig. 7E, F). Subsequently, it was shown that hE2/sE2 could significantly reduce the CB1 expression in neuronal cells and OVX mouse brain, as an important receptor in the retrograde endocannabinoid signaling pathway (Additional file 2: Fig. S2A-D) [13].

In the negative ion mode, compared with the OVX group, a total of 32 differential metabolites (3 down-regulated and 29 up-regulated) were identified in the OVX + sE2 group (Additional file 3: Fig. S3A). The KEGG enrichment analysis showed that the disorder of alanine, aspartate, and glutamate metabolism may be an important factor involved in the effects of sE2 on the brain of OVX mice, which was also a common cause related to the onset of depression (Additional file 3: Fig. S3B-F) [35].

## Discussion

**Fig. 8:**
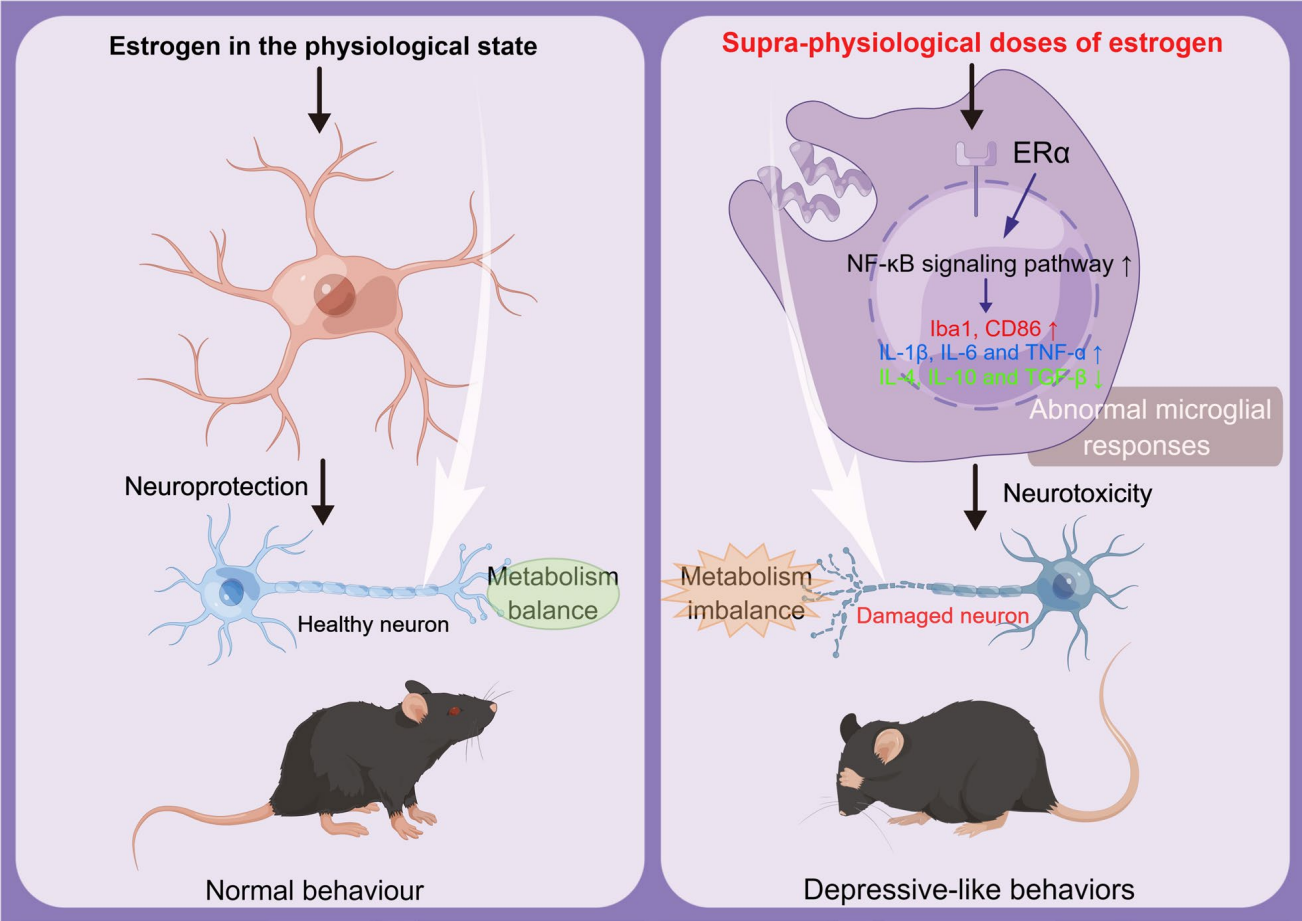
Under normal physiological conditions, E2 maintains microglial stability, and brain metabolic balance, thereby exerting its neuroprotective effects. The supraphysiological doses of estrogen (sE2) treatment activate the ERα/NF-κB pathway, promote the microglial response and brain metabolic imbalance, and then induce the neuroinflammation-mediated neuronal damage, ultimately resulting in depressive-like behaviors in mice

Our study was the first report demonstrating the risk of increased menopausal depression by sE2 in the treatment of ERT. Our results showed that sE2 could induce the microglial abnormal response through the ERα/NF-κB signaling pathway to cause neuronal damage and metabolic imbalance, ultimately aggravating the depressive-like behaviors of OVX mice. Furthermore, physiological doses of E2 maintain microglial stability, and brain metabolic balance, thereby exerting its neuroprotective effects (Fig. 8).

E2 is a steroid hormone that has been widely applied as a neuroprotective therapy for a variety of neurodegenerative and cerebrovascular disorders [36]. However, studies have shown that high doses of E2 used in the ERT increase the risk of endometrial cancer by 2–10 folds, while the patients treated with ERT are at a higher risk of developing ovarian cancer [37]. Several laboratory and clinical studies have reported that E2 showed no effect on these diseases in elderly postmenopausal women but increased the risk of the onset and even mortality of the patients of these diseases [38].

Xu et al. conducted animal studies to demonstrate that the administration of E2 effectively ameliorated the depressive behaviors through the inhibition of inflammation and the activation of indoleamino-2, 3-dioxygenase (IDO), ultimately regulating the levels of 5-hydroxytryptamine (5-HT) in the hippocampus [39]. However, the evidence for estrogenic antidepressant-like effects in humans is less conclusive compared to rodent studies, and in some cases, contradictory findings have been reported [40]. Therefore, the effective physiological doses of E2 supplementation for menopausal depression are still controversial. In fact, several studies have explored the medical effects of sE2. For example, Bronwyn et al. found that sE2 impaired the preestrus fear resolution in rats [41]. While Stephanie et al. found that high doses of E2 could prevent heart failure after myocardial infarction in rats [42].

In our study, the results revealed the potentially neurotoxic effect of sE2. To investigate the therapeutic effect of sE2 on OVX mice, the sE2 (0.5 mg/kg) was intraperitoneally injected into the OVX mice for 5 weeks. The results showed that sE2 treatment worsened depressive-like behaviors in OVX mice. It is necessary to note that more explorations of the cerebrotoxic effects of different E2 doses and durations of action are needed in the future. Although this study used different concentrations and time gradients to detect the effects of E2 on neurons and microglia in vitro, the in vivo experiment was a single-endpoint (single-dose) test and could not accurately reflect the effects of E2 on the mouse brain in a wider time range and concentration gradient. Furthermore, it is imperative to investigate the direct impact of ERT on the brains of healthy mice that have not undergone ovariectomy, considering the adverse cardiovascular and cerebrovascular effects associated with ERT. Consequently, it is noted that this study is deficient in experiments involving the administration of sE2 to sham-operated mice. Therefore, it would be worthwhile to include mouse models of primary ovarian failure or normal menopausal experience as research subjects in future studies, as the menopausal women differ from OVX mice in their physiological conditions.

Previous research showed that the exogenous E2 supplementation was frequently employed as a prevalent therapeutic approach for managing climacteric syndrome by sustaining the peripheral blood levels of estrogen at physiological ranges [1]. Typically, three methods are employed to maintain the physiological estrogen level in animals, i.e., subcutaneous or intraperitoneal injection of estradiol, the implantation of sustained-release capsules containing estradiol beneath the neck or oral gavage. However, the actual dose used was varied in different studies to maintain the normal physiological levels of estrogen. For instance, Zhu et al. administered a dosage of 0.36 mg/60-day E2 to address the abdominal obesity in OVX mice, while Adachi et al. employed a dosage of 0.05 mg/21-day E2 to treat psoriatic inflammation in OVX mice [44, 45]. Zhou et al. administered a dose of 0.3 mg/kg/d E2 orally to mice to improve the cognitive decline and depressive behavior induced by OVX [46]. Consequently, we contend that the decision to employ E2 as a physiological dose should be based on standard serological assessments. Therefore, in our investigation, a dosage of E2 was administered to obtain a twofold increase in the concentration of E2 in the peripheral blood of mice, which exceeded the physiological range.

Recent research has shown that depressed patients exhibited lower cognitive abilities compared to people who were not depressed [47]. It is generally believed that depression precedes the development of cognitive deficits [48]. Notably, we found that although sE2 aggravated depressive-like behaviors in OVX mice, it did not affect their short-term memory based on the results of the Y-maze test. This observation could be explained from two perspectives. First, the pathogenetic causes of both memory deficits and depression were different, i.e., depression was not necessarily sufficient to impair the memory function of the brain [49]. Second, the brain damage caused by sE2 was not sufficient to affect the neuronal circuits responsible for memory [50, 51].

Furthermore, studies have shown that elevated E2 levels lead to decreased levels of follicle-stimulating hormone (FSH) due to negative feedback from the pituitary gland [52, 53]. Recent studies have shown that elevated FSH levels are an important factor in the development of Alzheimer's disease in menopausal women [54]. Moreover, FSHR knockout mice showed significant depression and affective disorders [55, 56]. Given the association between FSH and depression, our study could not determine the explicit functions of sE2 in regulating depressive-like behaviors in mice without excluding the influence of FSH.

Epidemiological studies have shown that depression has a higher prevalence and morbidity in women than that in men, while female rats have a more active microglia phenotype, with longer and thicker protuberance and more intense phagocytosis [57, 58]. Our study showed that sE2 treatment increased the microglial activity in the cortical and hippocampal regions of OVX mice. Previous studied showed that increased levels of inflammatory cytokines in hippocampus contributed to E2 deficiency-induced depression-like behavior in rodents, while the ERβ mediated the estrogen modulation of neuroinflammation and affective behavior [59]. Therefore, given that microglia-mediated neuroinflammation is the important pathogenetic cause of depression, we hypothesized that the sE2 aggravated depressive-like behaviors in OVX mice by activating microglia [60]. This hypothesis was supported by the results of the in vitro experiments. Notably, in vitro, the low and high concentrations of E2 showed opposite effects on microglia which were already activated by LPS, with low and high concentrations of E2 inhibiting and enhancing proinflammatory response by microglia, respectively. Specifically, the E2 at twice its protective dose was sufficient to cause a pro-inflammatory response by microglia. This observation indicated that the effective dose range of E2 for neuroprotection was narrow, probably due to the different receptors and signaling pathways activated by different concentrations of E2. Previous reports have shown that E2 shows neuroprotective effects primarily through ERβ and CPER [61–63]. And ERβ mediates estrogen modulation of neuroinflammation and affective behavior [59]. In the microglia, the activation of NF-κB plays a critical role in the production of proinflammatory cytokines, leading to the neurotoxic effect [64]. Furthermore, our results of in vitro experiment showed that the hE2 promoted microglial proinflammatory response by activating the ERα/NF-κB signaling pathway, thus providing strong experimental evidence to support the above hypothesis.

Indeed, in a previous study, Fleischer et al. developed a novel ERβ agonist to enhance memory and reduce drug-induced vasodilation in OVX mice, i.e., the oral agent circumvented the adverse effects of overactivation of the ERα [65]. Therefore, we speculate that developing novel drugs to target estrogen receptors specifically, rather than by increasing E2 doses, would be a viable approach to treat menopausal depression in the future.

Notably, the E2 showed different effects on cell viability in primary cells (microglia and neuronal cells) compared to immortalized cell lines (BV2 and HT22 cells). On the one hand, the E2 showed a strong pro-proliferative effect on BV2 or HT22 cells regardless of the concentration. However, due to the low proliferative potential of primary microglia, the cell cycle tends to quiescence and senescence after differentiation [66]. On the other hand, the high concentration of E2 could decrease the viability of primary neurons by inducing metabolic disorders, and HT22 cells themselves showed a strong regulatory ability to maintain the metabolic balance. In addition, in vitro models do not fully represent in vivo conditions. Therefore, further studies are still needed to confirm the security and the specific mechanisms in vivo of sE2.

Finally, the untargeted metabolomics results showed that the sE2 caused the imbalance of multiple metabolites involved in glycerophospholipid metabolism, retrograde endocannabinoid signaling, and so on, which are associated with depression. These results are consistent with the findings of Zhang et al. [6], showing that the long-term ERT treatment promotes CB1 ubiquitination in the brain of OVX mice, ultimately causing the fear extinction disorder [67]. Previous studies have shown that gut microbes induce depression by regulating glycerophospholipid metabolism, people with sleep deprivation and depression have abnormal glycerophospholipid metabolism, while abnormal glycerophospholipid metabolism also occurs in the brain of the depression rat model [68–70]. However, the molecular mechanisms regulating the participation of these metabolites in the above metabolic pathways remain unclear. Further studies are necessary to confirm whether the metabolic imbalance caused by sE2 is limited to neuronal cells, which also affects the metabolisms of microglia and astrocytes.

In conclusion, the sE2 induced depression in OVX mice in various ways, including the abnormal response of microglia and metabolic imbalance of neuronal cells. This study has revealed the potential risks and the underlying mechanisms of the application of sE2 in ERT in the treatment of menopausal depression.

### Supplementary Information

**Additional file 1: Figure S1. **Exogenous estrogen supplementation significantly increased peripheral blood estrogen levels in ovariectomized mice. (A) A Schematic illustration of the workflow of animal experiments. (B) Serum 17β-Estradiol levels in all groups of mice. Students t-test is performed to determine the significant difference based on P < 0.05 (*), P < 0.01 (**), and P < 0.001 (***), respectively, in comparison to the sham group (as indicated by black asterisks and “ns”). ns: not significant difference. Data are presented as mean ± the standard deviation (SD) of at least four animals per group.

**Additional file 2: Figure S2. **Cannabinoid receptor 1 (CB1) inhibited by hE2/sE2 in primary neuron cells and OVX mouse brain. (A) Primary neuron cells fixed and immunostained for CB1 (red). Bar = 30 µm. Cells are incubated with E2 of 0 nmol/L to 3200 nmol/L for 24 h. (B) Mean fluorescence intensity of CB1 expressed as a relative change in comparison with untreated cells. (C) The mRNA levels of CB1 in primary neurons treated with E2 of different concentrations. (D) RNA levels of CB1 in hippocampus and cortex of mice in each group. Students t-test is performed to determine the significant difference based on P < 0.05 (*), P < 0.01 (**), and P < 0.001 (***), respectively, in comparison to the sham or control groups as indicated by black asterisks or “ns” and to the OVX group as indicated by red asterisks. ns: no significant difference. Data are presented as mean ± standard deviation (SD). Each experiments is repeated independently twice. Data are based on a minimum of 10 animals in each group.

**Additional file 3: Figure S3. **Analysis of non‐targeted metabolomics of brains from sE2‐treated OVX mice compared to vehicle‐treated OVX mice under negative ions. (A) Heatmap of 48 significantly changed metabolites based on untargeted metabolomics. (B) KEGG enrichment analysis of the differential metabolites. The X-axis indicates the number of annotated metabolites under a certain pathway as a percentage of all annotated metabolites. (C) Matchstick analysis of the differential metabolites. Red boxes indicate metabolites related to glycerophospholipid metabolism and retrograde endocannabinoid signaling. ANOVA is performed to determine the significant difference based on P < 0.05 (*), P < 0.01 (**), and P < 0.001 (***), respectively. (D) Pathway enrichment of differential metabolites. (E) Network analysis of the differential metabolites. (F) Heatmap of correlation analysis of differential metabolites. Each group contains a total of 5 animals.

**Additional file 4: Table S1.** Primers and their sequences used for the quantitative real time PCR. **Table S2.** Primers and their sequences of siRNA duplexes for gene knockdown experiments.

**Additional file 5：** Original western blots.

## Data Availability

The data used and/or analyzed during the study are available from the corresponding author on reasonable request.
