# Supplementary figures and images for: Supraphysiologic doses of 17β-estradiol aggravate depression-like behaviors in ovariectomized mice possibly via regulating microglial responses and brain glycerophospholipid metabolism
Source: J Neuroinflammation. 2023 Sep 7;20:204. doi: 10.1186/s12974-023-02889-5 (PMC10485970; doi:10.1186/s12974-023-02889-5)

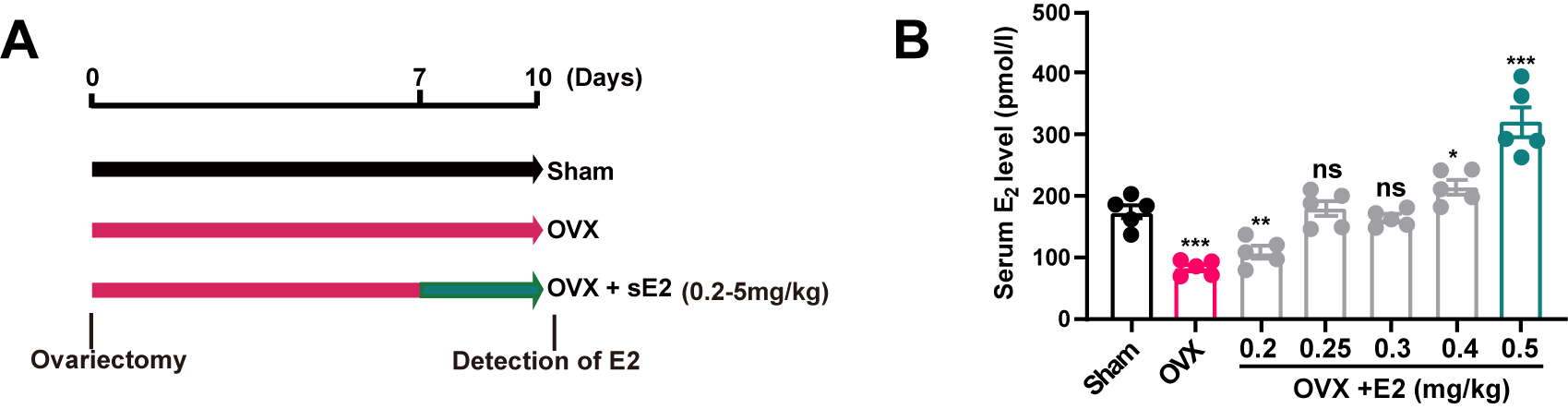

Supplement: Supplementary file 1 — Additional file 1: Figure S1. Exogenous estrogen supplementation significantly increased peripheral blood estrogen levels in ovariectomized mice. (A) A Schematic illustration of the workflow of animal experiments. (B) Serum 17β-Estradiol levels in all groups of mice. Students t-test is performed to determine the significant difference based on P < 0.05 (*), P < 0.01 (**), and P < 0.001 (***), respectively, in comparison to the sham group (as indicated by black asterisks and “ns”). ns: not significant difference. Data are presented as mean ± the standard deviation (SD) of at least four animals per group. [file 12974_2023_2889_MOESM1_ESM.tif]

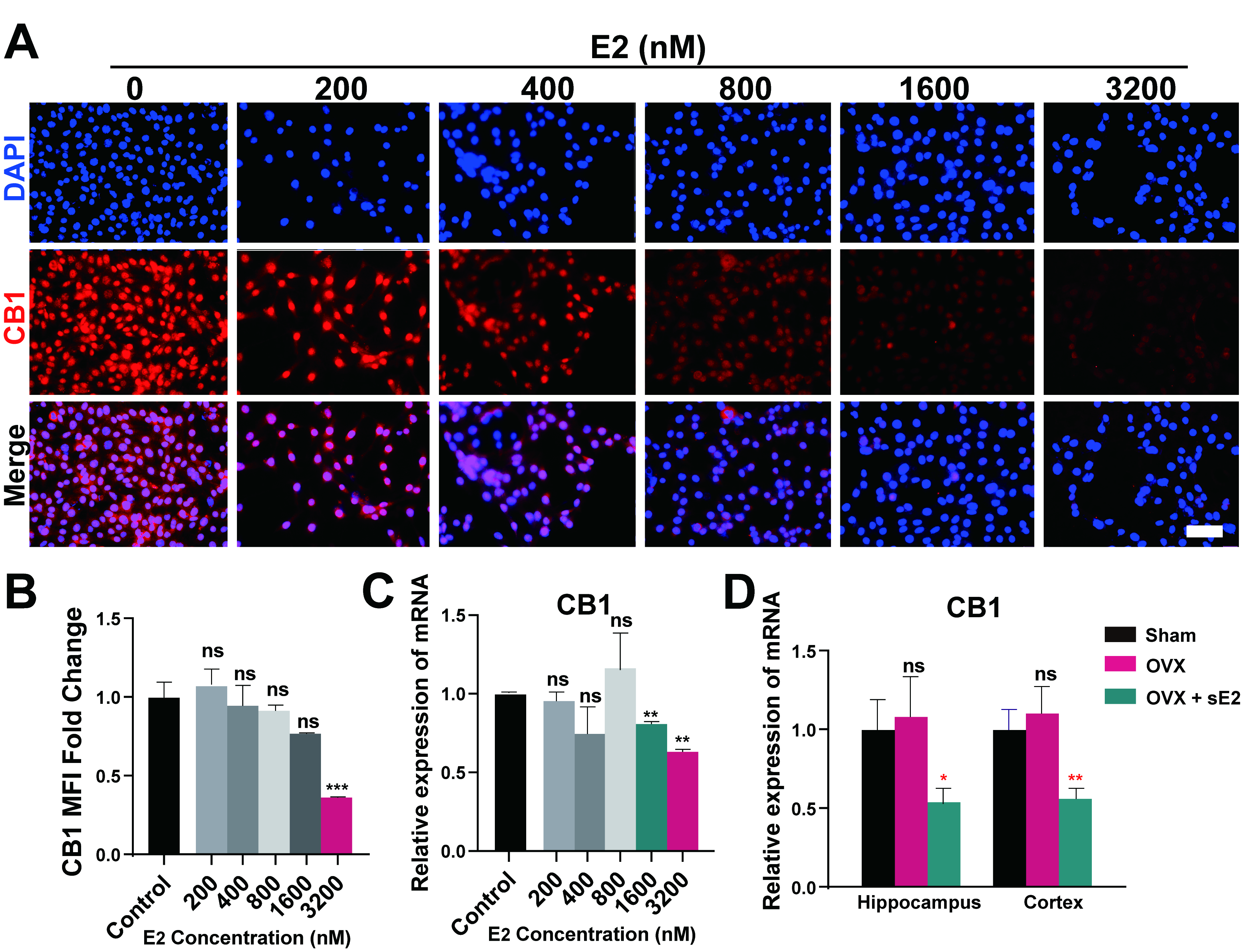

Supplement: Supplementary file 2 — Additional file 2: Figure S2. Cannabinoid receptor 1 (CB1) inhibited by hE2/sE2 in primary neuron cells and OVX mouse brain. (A) Primary neuron cells fixed and immunostained for CB1 (red). Bar = 30 µm. Cells are incubated with E2 of 0 nmol/L to 3200 nmol/L for 24 h. (B) Mean fluorescence intensity of CB1 expressed as a relative change in comparison with untreated cells. (C) The mRNA levels of CB1 in primary neurons treated with E2 of different concentrations. (D) RNA levels of CB1 in hippocampus and cortex of mice in each group. Students t-test is performed to determine the significant difference based on P < 0.05 (*), P < 0.01 (**), and P < 0.001 (***), respectively, in comparison to the sham or control groups as indicated by black asterisks or “ns” and to the OVX group as indicated by red asterisks. ns: no significant difference. Data are presented as mean ± standard deviation (SD). Each experiments is repeated independently twice. Data are based on a minimum of 10 animals in each group. [file 12974_2023_2889_MOESM2_ESM.tif]

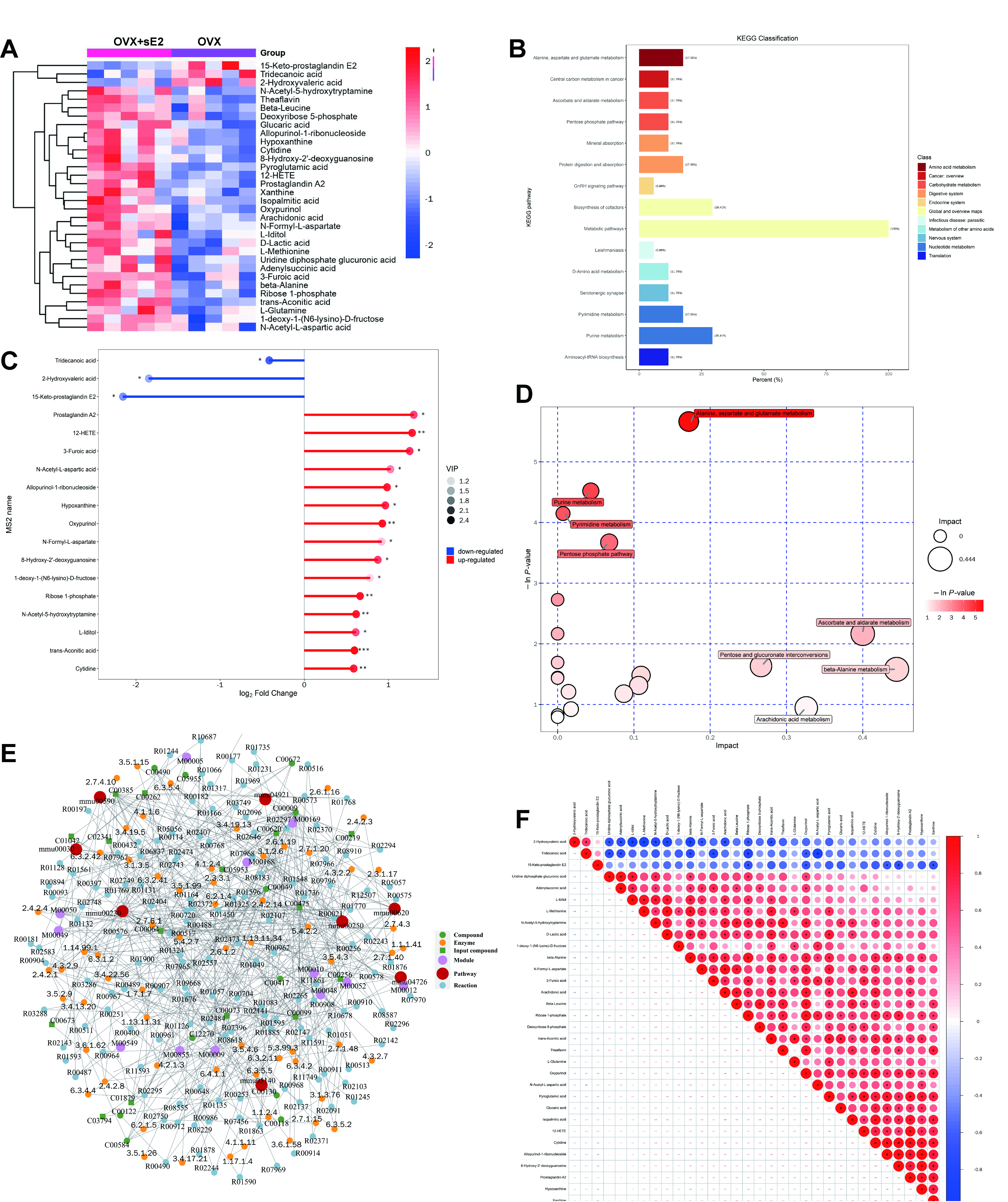

Supplement: Supplementary file 3 — Additional file 3: Figure S3. Analysis of non‐targeted metabolomics of brains from sE2‐treated OVX mice compared to vehicle‐treated OVX mice under negative ions. (A) Heatmap of 48 significantly changed metabolites based on untargeted metabolomics. (B) KEGG enrichment analysis of the differential metabolites. The X-axis indicates the number of annotated metabolites under a certain pathway as a percentage of all annotated metabolites. (C) Matchstick analysis of the differential metabolites. Red boxes indicate metabolites related to glycerophospholipid metabolism and retrograde endocannabinoid signaling. ANOVA is performed to determine the significant difference based on P < 0.05 (*), P < 0.01 (**), and P < 0.001 (***), respectively. (D) Pathway enrichment of differential metabolites. (E) Network analysis of the differential metabolites. (F) Heatmap of correlation analysis of differential metabolites. Each group contains a total of 5 animals. [file 12974_2023_2889_MOESM3_ESM.tif]

**Figure 3E**

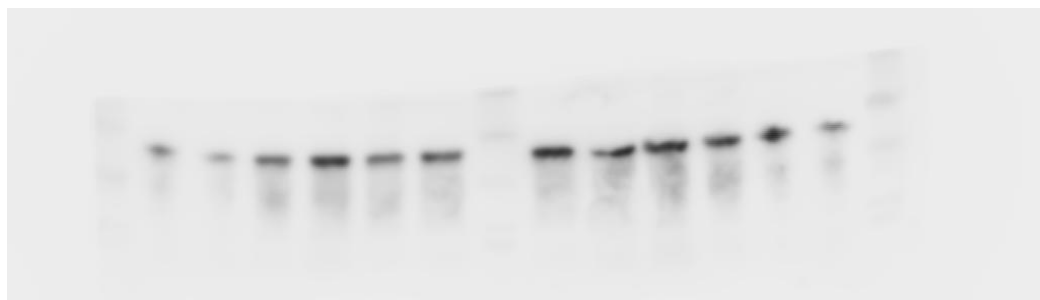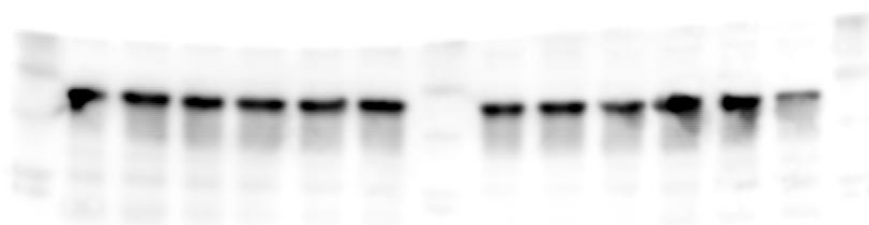

**Figure 4B and F**

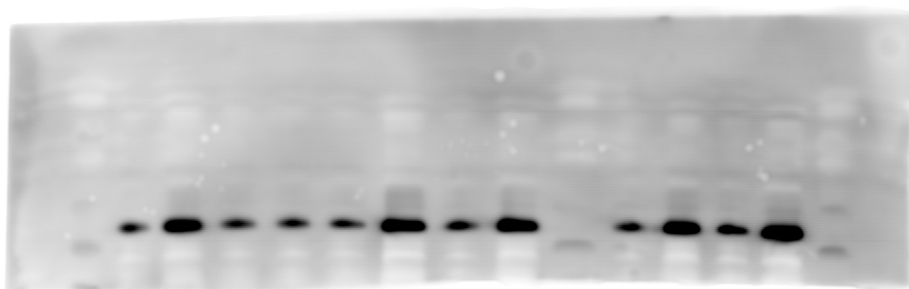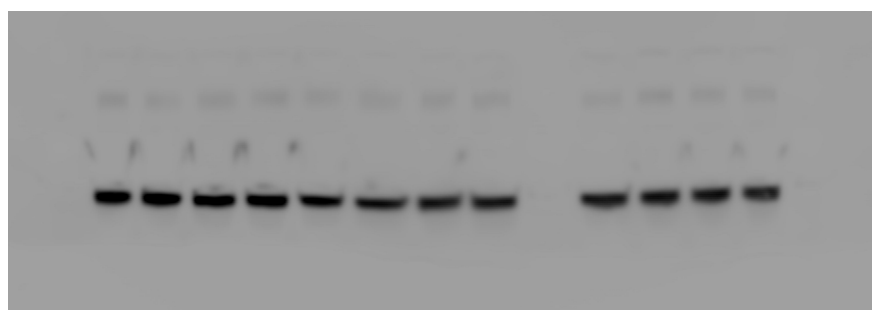

Figure 4C

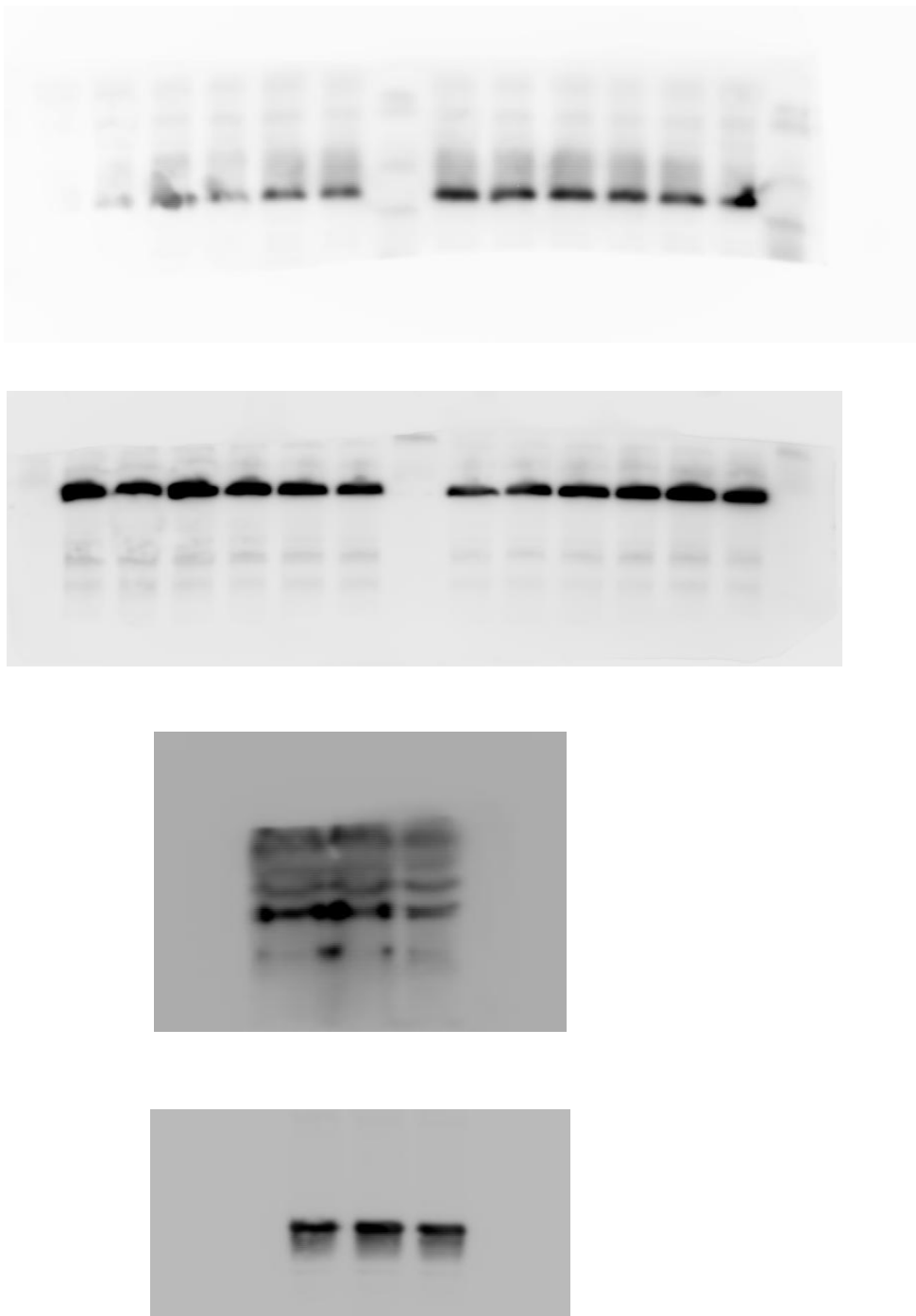

Supplement: Supplementary file 5 — Additional file 5： Original western blots. [file 12974_2023_2889_MOESM5_ESM.pdf]
