# Supplementary material for: Supraphysiologic doses of 17β-estradiol aggravate depression-like behaviors in ovariectomized mice possibly via regulating microglial responses and brain glycerophospholipid metabolism
Source: J Neuroinflammation. 2023 Sep 7;20:204. doi: 10.1186/s12974-023-02889-5 (PMC10485970; doi:10.1186/s12974-023-02889-5)
Supplement: Supplementary file 4 — Additional file 4: Table S1. Primers and their sequences used for the quantitative real time PCR. Table S2. Primers and their sequences of siRNA duplexes for gene knockdown experiments. [file 12974_2023_2889_MOESM4_ESM.docx]

**Table S1.** Primers and their sequences used for the quantitative real time PCR.

| **Gene** | **Forward primer (5’ to 3’)** | **Reverse primer (5’ to 3’)** |
| --- | --- | --- |
| GAPDH | CAATGACCCCTTCATTGACC | TGGACTCCACGACGTACTCA |
| IL-10 | ACTTTAAGGGTTACCTGGGTTGC | TCACATGCGCCTTGATGTCTG |
| TNF-α | CAGAGGGAAGAGTTCCCCAG | CCTCAGCTTGAGGGTTTGCTAC |
| IL-1β | TGGTGTGTGACGTTCCCATTA | CAGCACGAGGCTTTTTTGTTG |
| IL-6 | ACAACCACGGCCTTCCCTACTT | CACGATTTCCCAGAGAACATGTG |
| IL-4 | CATCGGCATTTTGAACGAGGTCA | CTTATCGATGAATCCAGGCATCG |
| TGF-β | CACCTGCAAGACCATCGAC | TGGCGAGCCTTAGTTTGGAC |
| ERα | CACCAGATCCAAGGGAA | CGGCGTTGAACTCGTAG |
| ERβ | GACTGTAGAACGGTGTGGTCATCAA | CTGTGAGGTAGGAATGCGAAAC |
| GPER | TTCCTCACCTGGATGAGCTT | CCTGACATCAGCAAAGCAGA |
| CB1 | CCTGACATCAGCAAAGCAGA | CATTGGGGCTGTCTTTACGG |

**Table S2.** Primers and their sequences of siRNA duplexes for gene knockdown experiments.

| **Gene** | **Forward primer (5’ to 3’)** | **Reverse primer (5’ to 3’)** |
| --- | --- | --- |
| ERα_BV2 | CCAGAAUGGCCGAGAGAGATT | UCUCUCUCGGCCAUUCUGGTT |
| ERα_Primary microglia | GGGAGAAUGUUGAAGCACATT | UGUGCUUCAACAUUCUCCCTT |
| ERβ_BV2 | CGCAAGACAUGGAGAUCAATT | UUGAUCUCCAUGUCUUGCGTT |
| ERβ_Primary microglia | ACAAGGAACUGGUGCACAUTT | AUGUGCACCAGUUCCUUGUTT |
| GPER_BV2 | GGAGAGACCUUCAGGGACATT | UGUCCCUGAAGGUCUCUCCTT |
| GPER_Primary microglia | GGAUGAGCUUCGACAGGUATT | UACCUGUCGAAGCUCAUCCTT |
| Negative control | UUCUCCGAACGUGUCACGUTT | ACGUGACACGUUCGGAGAATT |
| Positive control | UUGAUGACAAGCUUCCCAUUCUTT | AGAAUGGGAAGCUUGUCAUCAATT |
